# Supplementary material for: Clinically Feasible White Matter Fiber Tractography in Peritumoral Zones With Cerebral Vasogenic Edema
Source: Magn Reson Med. 2026 Feb 22;96(1):287–301. doi: 10.1002/mrm.70314 (PMC13156450; doi:10.1002/mrm.70314)
Supplement: Supplementary file 1 — Table S1: Microstructure parameters used for generating ODF‐dictionaries. Figure S1: The relative overlap between tractography and edema (mean and standard deviation) in the fully sampled (green) and the subsampled clinically feasible data sets (purple) calculated for all the reconstructed fascicles. ODF‐FP is compared with the best‐performing reference method, that is, FSL in the fully sampled and SS3T‐CSD in the subsampled data set. The p values of the covariates (patient's age and sex, tumor hemisphere and tumor type) are given on the right side. Figure S2: Box plots of the relative overlap between Corticobulbar Tract (CBT) and the cortical area activated during the lip puckering functional MRI (fMRI) task. Tractography was produced from the fully sampled (green) or the subsampled clinically feasible diffusion MRI (purple). The mean and standard deviations of the normalized True positive rates, Dice scores, and 95% Hausdorff distances are given above the respective box whiskers. ODF‐FP is compared with the best‐performing reference method, i.e., FSL in the fully sampled and SS3T‐CSD in the subsampled data set. The p values of the covariates (patient's age and sex, tumor hemisphere and tumor type) are given on the right side. Figure S3: Box plots of the relative overlap between Arcuate Fasciculus (AF) and the cortical area activated during the reading functional MRI (fMRI) task. Tractography was produced from the fully sampled (green) or the subsampled clinically feasible diffusion MRI (purple). The mean and standard deviations of the normalized True positive rates, Dice scores, and 95% Hausdorff distances are given above the respective box whiskers. ODF‐FP is compared with the best‐performing reference method, i.e., FSL in the fully sampled and SS3T‐CSD in the subsampled data set. The p values of the covariates (patient's age and sex, tumor hemisphere and tumor type) are given on the right side. Figure S4: Box plots of the relative overlap between Superior Longit [file MRM-96-287-s001.pdf]

SUPPLEMENTARY MATERIAL

Clinically feasible white matter fiber tractography in peritumoral zones with cerebral vasogenic edema

Patryk Filipiak[1] | Timothy M. Shepherd[1] | Kamri Clarke[1] | Gaia Ressa[1,2] | Dimitris G. Placantonakis[3] | Fernando E. Boada[4] | Steven H. Baete[1]

[1]Center for Advanced Imaging Innovation and Research (CAI^{2}R), Department of Radiology, NYU Langone Health, New York, NY, USA
[2]Neuroradiology Unit, IRCCS Humanitas Research Hospital, Rozzano, Milan, Italy
[3]Department of Neurosurgery, Perlmutter Cancer Center, Neuroscience Institute, Kimmel Center for Stem Cell Biology, NYU Langone Health, New York, NY, USA
[4]Radiological Sciences Laboratory and Molecular Imaging Program at Stanford, Department of Radiology, Stanford University, Stanford, CA, USA

Correspondence
Patryk Filipiak, Department of Radiology, NYU Langone Health.
Email: patryk.filipiak@nyulangone.org

Present Address
660 1st Ave, New York, NY 10016, USA

Funding Information
National Institutes of Health; Grant/Award Numbers: R01 EB028774, R01 EB029306, P41 EB017183.

Summary

Purpose: In diffusion MRI, vasogenic edema manifests as a major fraction of isotropic water that dilutes the anisotropic intra-axonal portion of the signal. Many tractography algorithms mistake vasogenic edema for the white matter boundary and terminate tracking to prevent producing spurious streamlines. As a result, visual representations of fascicles traversing edema are often compromised, limiting the clinical utility of tractography.
Methods: We address this hurdle with ODF-Fingerprinting (ODF-FP) — a dictionary-based fiber reconstruction algorithm that accommodates variability of neural tissue. By adding a regularization term to the ODF-FP matching formula, we counterbalance the drop of diffusion anisotropy in edematous regions to improve white matter fiber identification. In 19 glioma cases with significant peritumoral vasogenic edema, we quantify the volume of the reconstructed white matter tracts immersed in edema, then we use the cortical regions activated during task-based functional MRI as validation for tractography. To assess the potential for clinical translation, we additionally test the performance of ODF-FP on subsampled single-shell diffusion-weighted images, which contemporary clinical scanners can acquire within a few minutes.
Results: Our approach produces high volumes of streamlines traversing vasogenic edema and reaches high overlap with the cortical regions activated at task-based fMRI, significantly outperforming common fiber reconstruction methods in the clinically feasible data set.
Conclusion: ODF-FP proves effective on research and clinical quality dMRI, which offers an opportunity for application in neurosurgery.

KEYWORDS:
vasogenic edema, tractography, diffusion MRI, ODF-fingerprinting, brain tumor, peritumoral zone, surgical planning, functional MRI

Received 26 April 2016; | Revised 6 June 2016; | Accepted 6 June 2016

DOI: xxx/xxxx

How to cite this article: Filipiak P., Shepherd T.M., Clarke K., Ressa G., Placantonakis D.G., Boada F.E., and Baete S.H. (2026), Clinically feasible white matter fiber tractography in peritumoral zones with cerebral vasogenic edema, *Magn. Reson. Med.*

Fraction sizes		Range
p_{fib}	fiber fraction	$[0, 1]$
p_{iso}	isotropic fraction	$[0, 1]$
f_{in}	intra-axonal fraction	$[0, 1]$
**Diffusivities**		**Range [m^2/s]**
D_a	intra-axonal	$[1.5, 2.5] \cdot 10^{-9}$
$D_e^{\parallel}$	extra-axonal parallel	$[1.5, 2.5] \cdot 10^{-9}$
$D_e^{\perp}$	extra-axonal perpendicular	$[0.5, 1.5] \cdot 10^{-9}$
D_{iso}	isotropic	$[2.0, 3.0] \cdot 10^{-9}$

Supplementary Table S1. Microstructure parameters used for generating ODF-dictionaries.

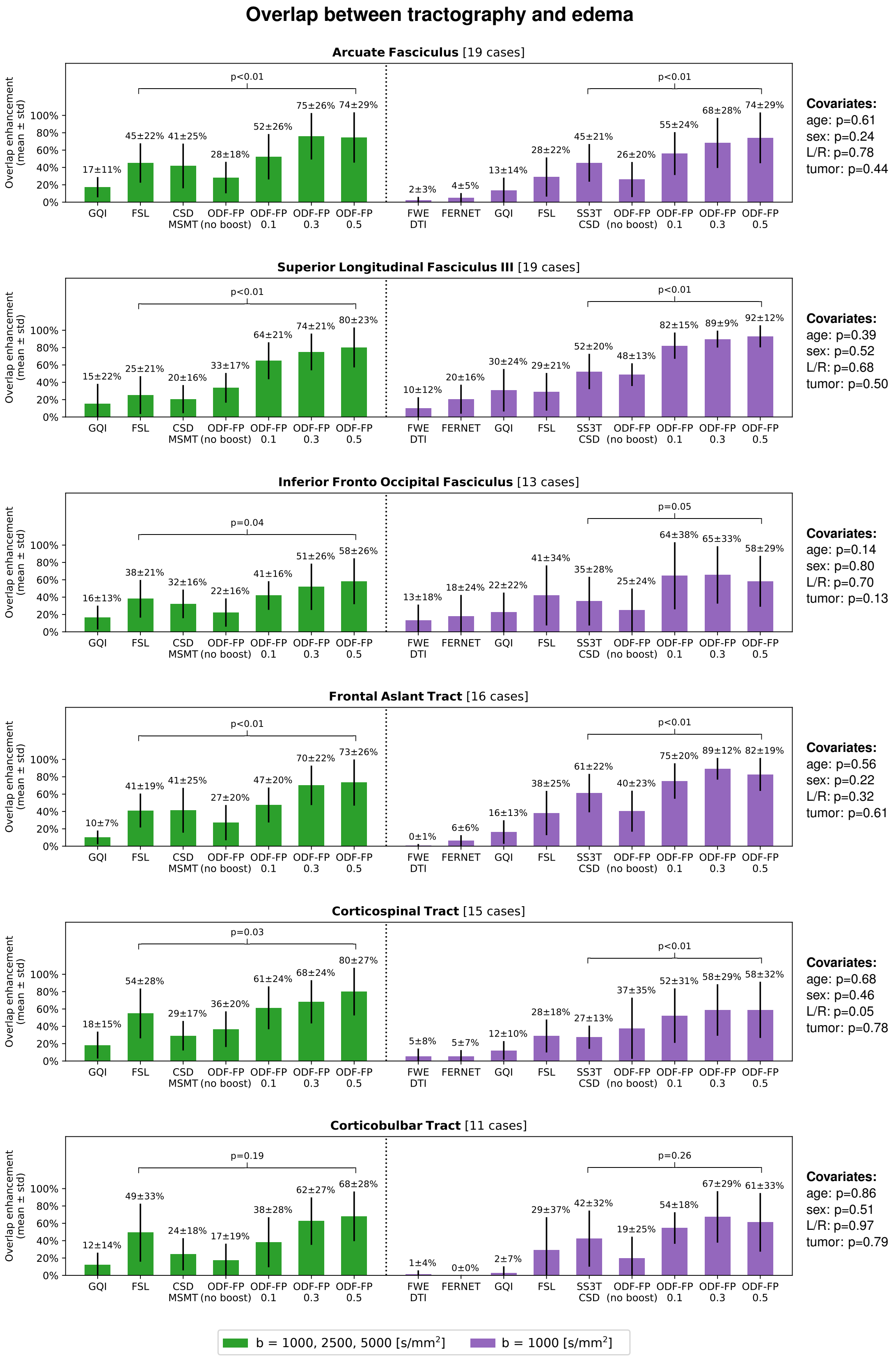

Supplementary Figure S1. The relative overlap between tractography and edema (mean and standard deviation) in the fully sampled (green) and the subsampled data sets (purple) calculated for all the reconstructed fascicles. ODF-FP is compared with the best-performing reference method, i.e., FSL in the fully sampled and SS3T-CSD in the subsampled data set. The p-values of the covariates (patient's age and sex, tumor hemisphere and tumor type) are given on the right side.

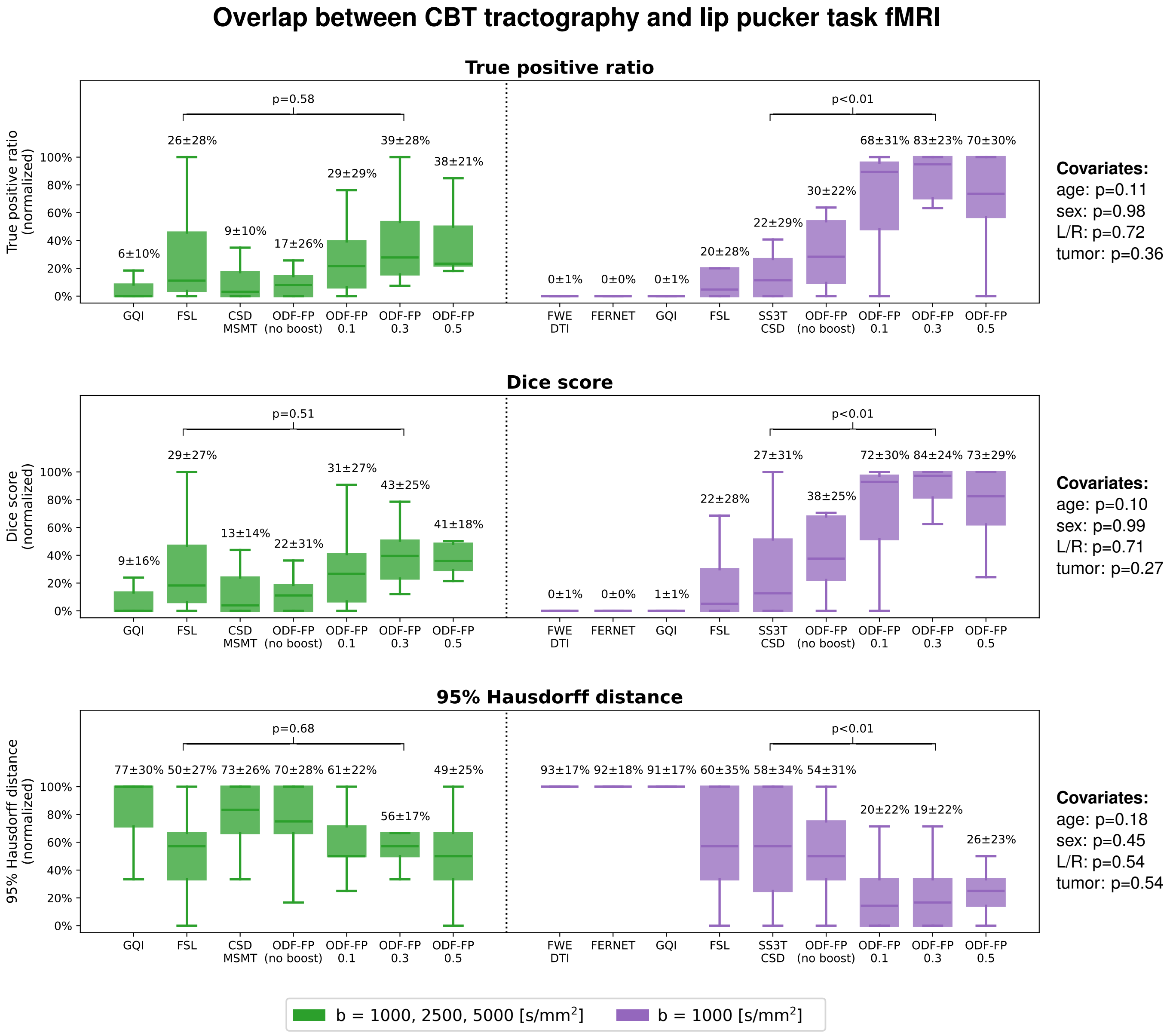

Supplementary Figure S2. Box plots of the relative overlap between Corticobulbar Tract (CBT) and the cortical area activated during the lip puckering functional MRI (fMRI) task. Tractography was produced from the fully sampled (green) or the subsampled clinically-feasible diffusion MRI (purple). The mean and standard deviations of the normalized True positive rates, Dice scores, and 95% Hausdorff distances are given above the respective box whiskers. ODF-FP is compared with the best-performing reference method, i.e., FSL in the fully sampled and SS3T-CSD in the subsampled data set. The p-values of the covariates (patient's age and sex, tumor hemisphere and tumor type) are given on the right side.

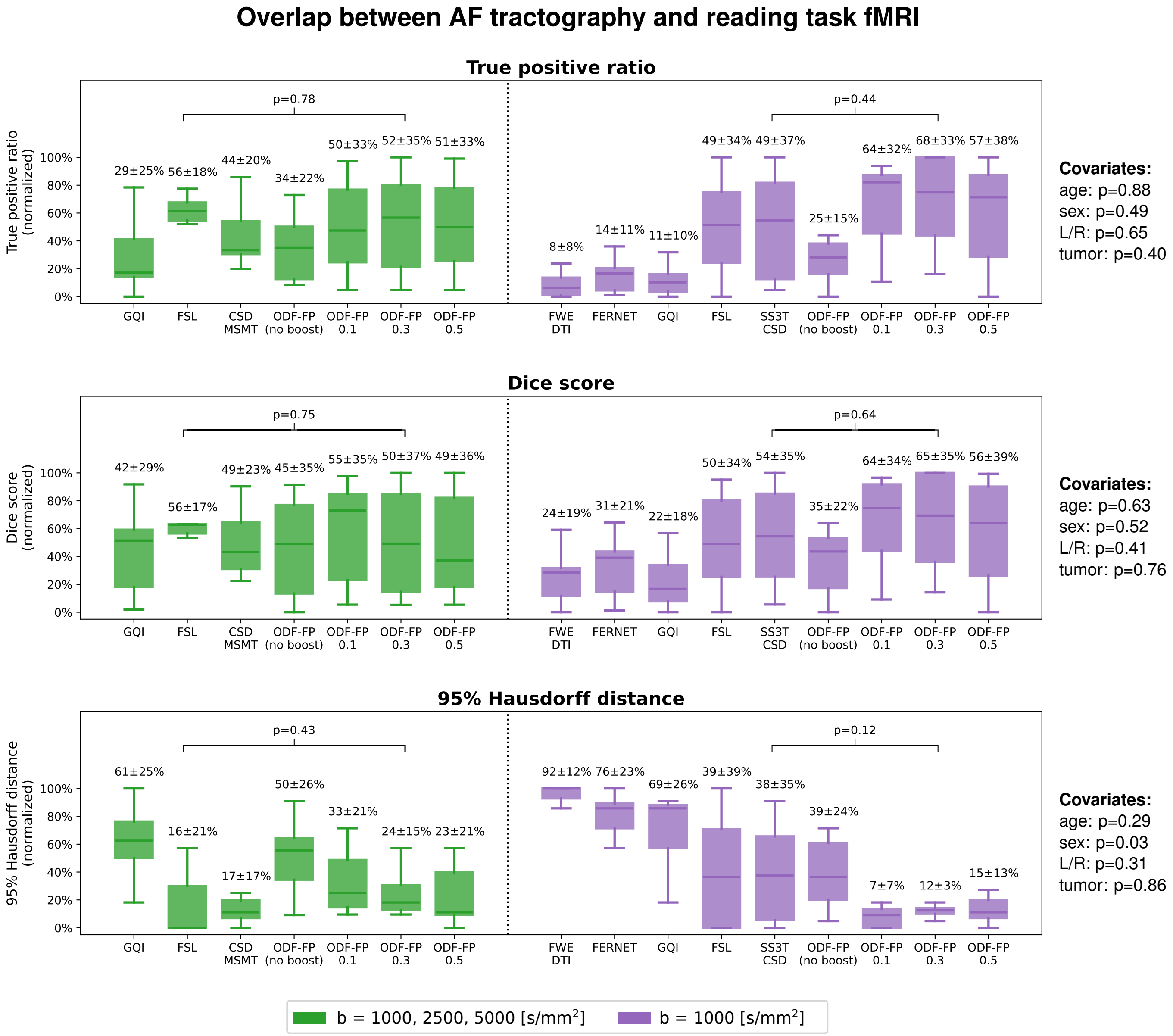

Supplementary Figure S3. Box plots of the relative overlap between Arcuate Fasciculus (AF) and the cortical area activated during the reading functional MRI (fMRI) task. Tractography was produced from the fully sampled (green) or the subsampled clinically-feasible diffusion MRI (purple). The mean and standard deviations of the normalized True positive rates, Dice scores, and 95% Hausdorff distances are given above the respective box whiskers. ODF-FP is compared with the best-performing reference method, i.e., FSL in the fully sampled and SS3T-CSD in the subsampled data set. The p-values of the covariates (patient's age and sex, tumor hemisphere and tumor type) are given on the right side.

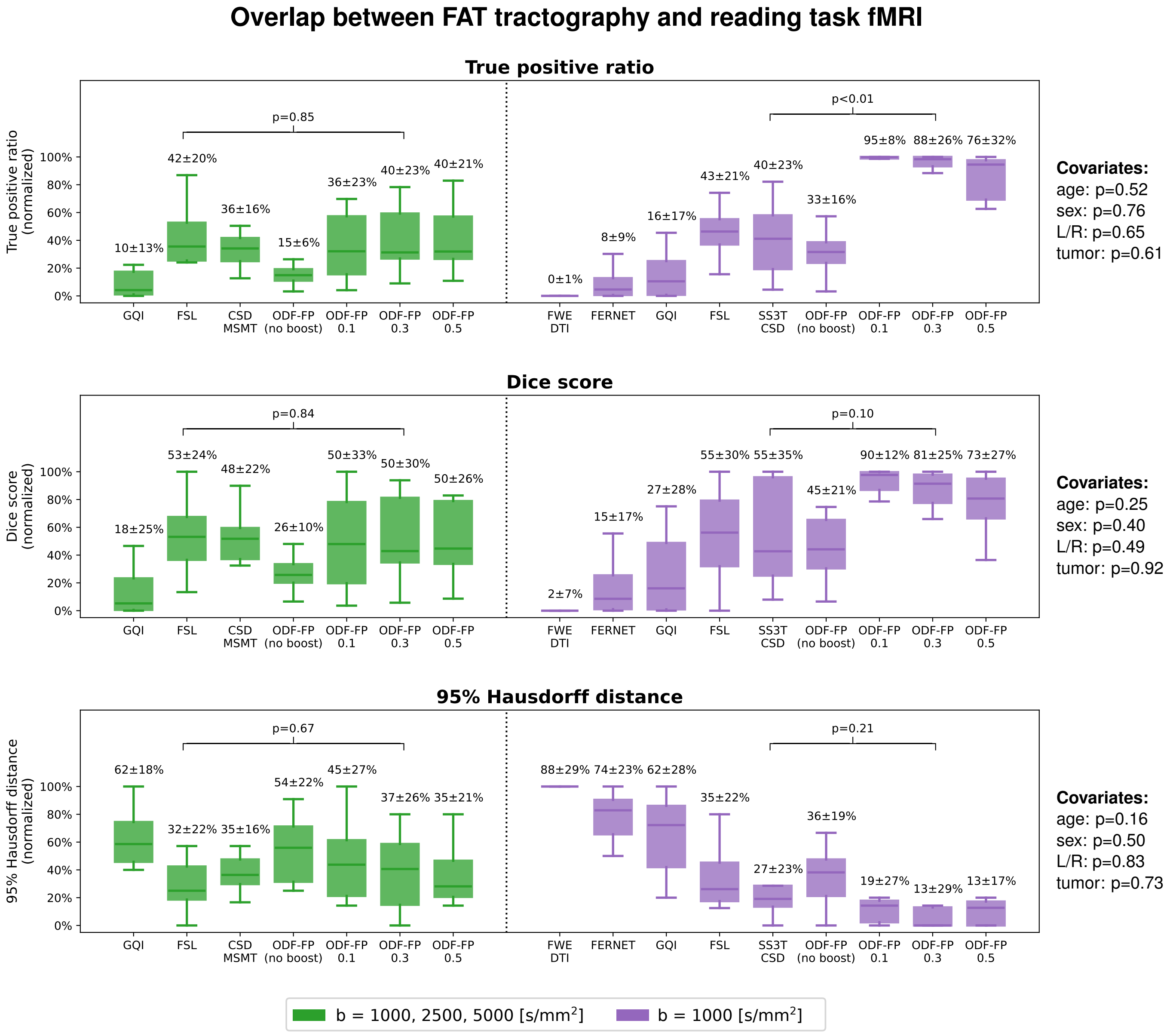

Supplementary Figure S4. Box plots of the relative overlap between Superior Longitudinal Fasciculus III (SLF3) and the cortical area activated during the reading functional MRI (fMRI) task. Tractography was produced from the fully sampled (green) or the subsampled clinically-feasible diffusion MRI (purple). The mean and standard deviations of the normalized True positive rates, Dice scores, and 95% Hausdorff distances are given above the respective box whiskers. ODF-FP is compared with the best-performing reference method, i.e., FSL in the fully sampled and SS3T-CSD in the subsampled data set. The p-values of the covariates (patient's age and sex, tumor hemisphere and tumor type) are given on the right side.

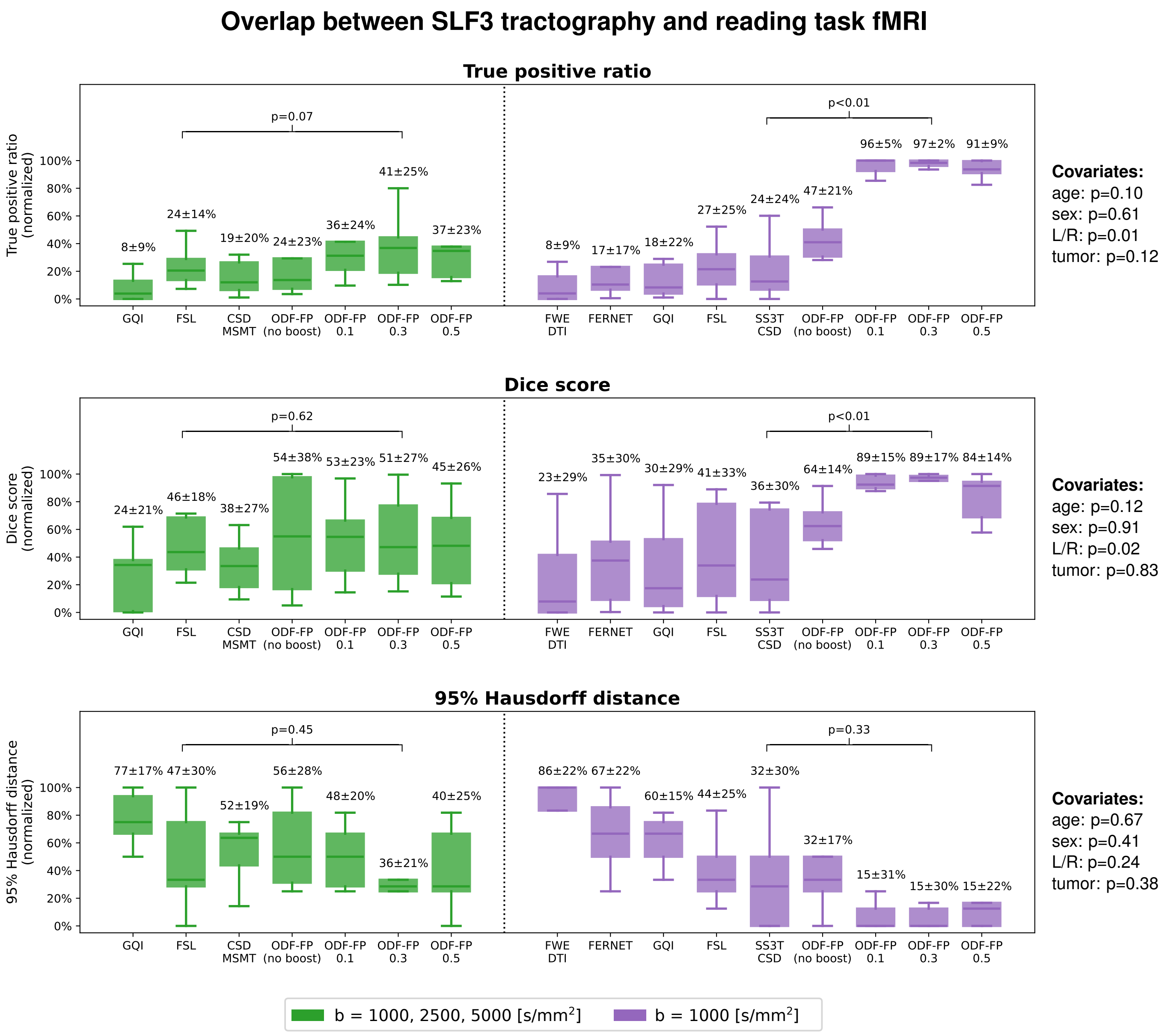

Supplementary Figure S5. Box plots of the relative overlap between Frontal Aslant Tract (FAT) and the cortical area activated during the reading functional MRI (fMRI) task. Tractography was produced from the fully sampled (green) or the subsampled clinically-feasible diffusion MRI (purple). The mean and standard deviations of the normalized True positive rates, Dice scores, and 95% Hausdorff distances are given above the respective box whiskers. ODF-FP is compared with the best-performing reference method, i.e., FSL in the fully sampled and SS3T-CSD in the subsampled data set. The p-values of the covariates (patient's age and sex, tumor hemisphere and tumor type) are given on the right side.

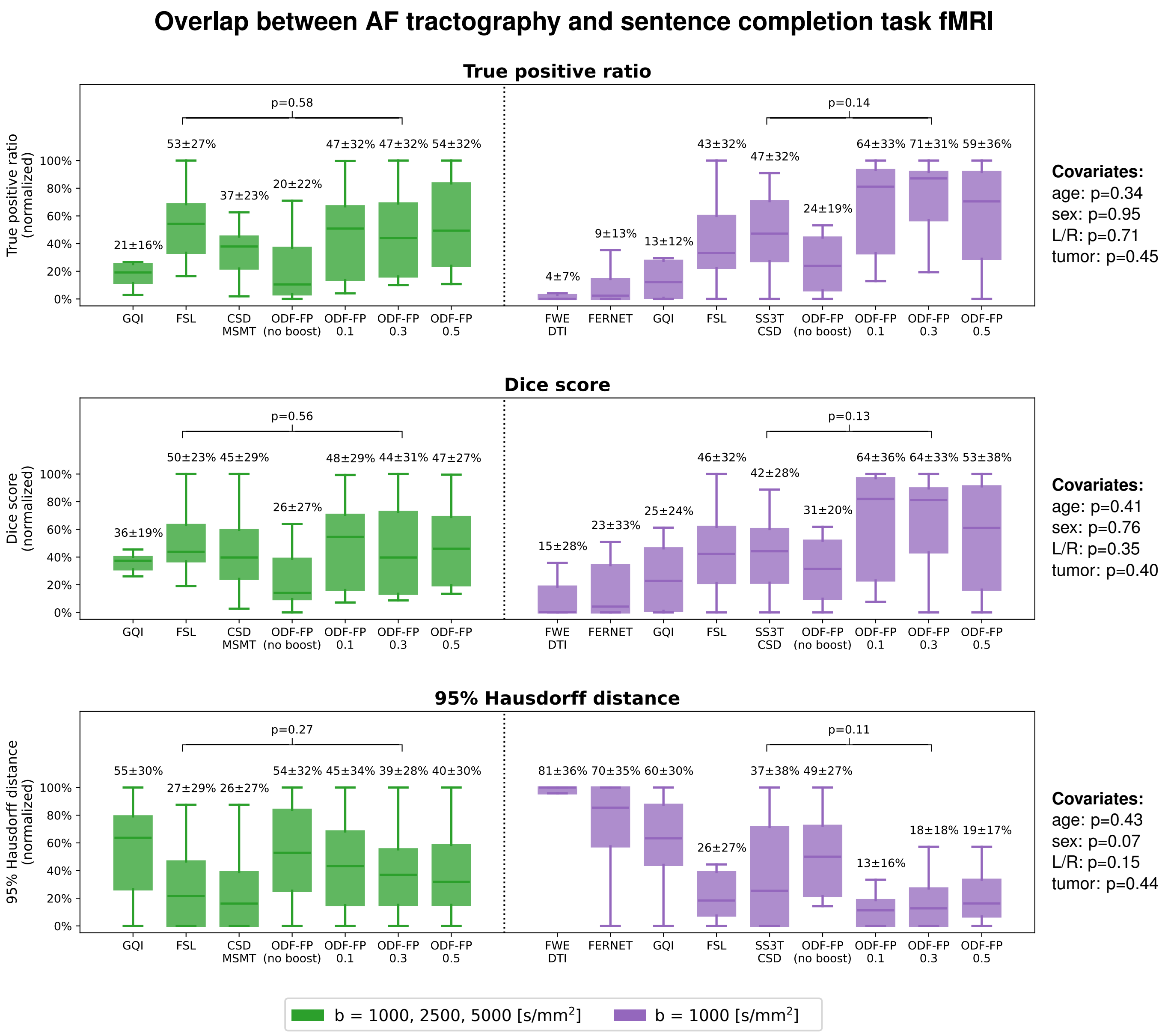

Supplementary Figure S6. Box plots of the relative overlap between Arcuate Fasciculus (AF) and the cortical area activated during the sentence completion functional MRI (fMRI) task. Tractography was produced from the fully sampled (green) or the subsampled clinically-feasible diffusion MRI (purple). The mean and standard deviations of the normalized True positive rates, Dice scores, and 95% Hausdorff distances are given above the respective box whiskers. ODF-FP is compared with the best-performing reference method, i.e., FSL in the fully sampled and SS3T-CSD in the subsampled data set. The p-values of the covariates (patient's age and sex, tumor hemisphere and tumor type) are given on the right side.

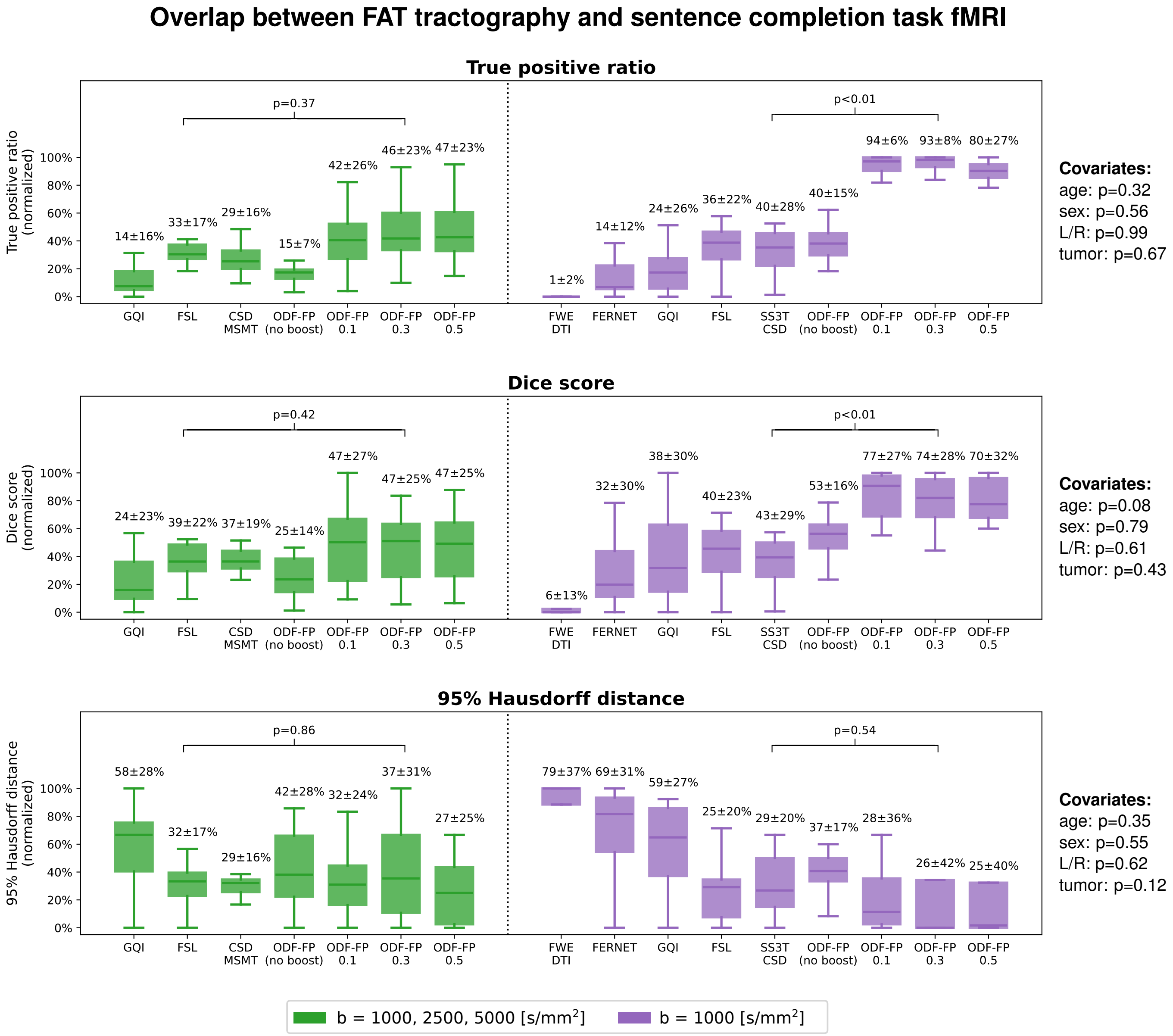

Supplementary Figure S7. Box plots of the relative overlap between Superior Longitudinal Fasciculus III (SLF3) and the cortical area activated during the sentence completion functional MRI (fMRI) task. Tractography was produced from the fully sampled (green) or the subsampled clinically-feasible diffusion MRI (purple). The mean and standard deviations of the normalized True positive rates, Dice scores, and 95% Hausdorff distances are given above the respective box whiskers. ODF-FP is compared with the best-performing reference method, i.e., FSL in the fully sampled and SS3T-CSD in the subsampled data set. The p-values of the covariates (patient's age and sex, tumor hemisphere and tumor type) are given on the right side.

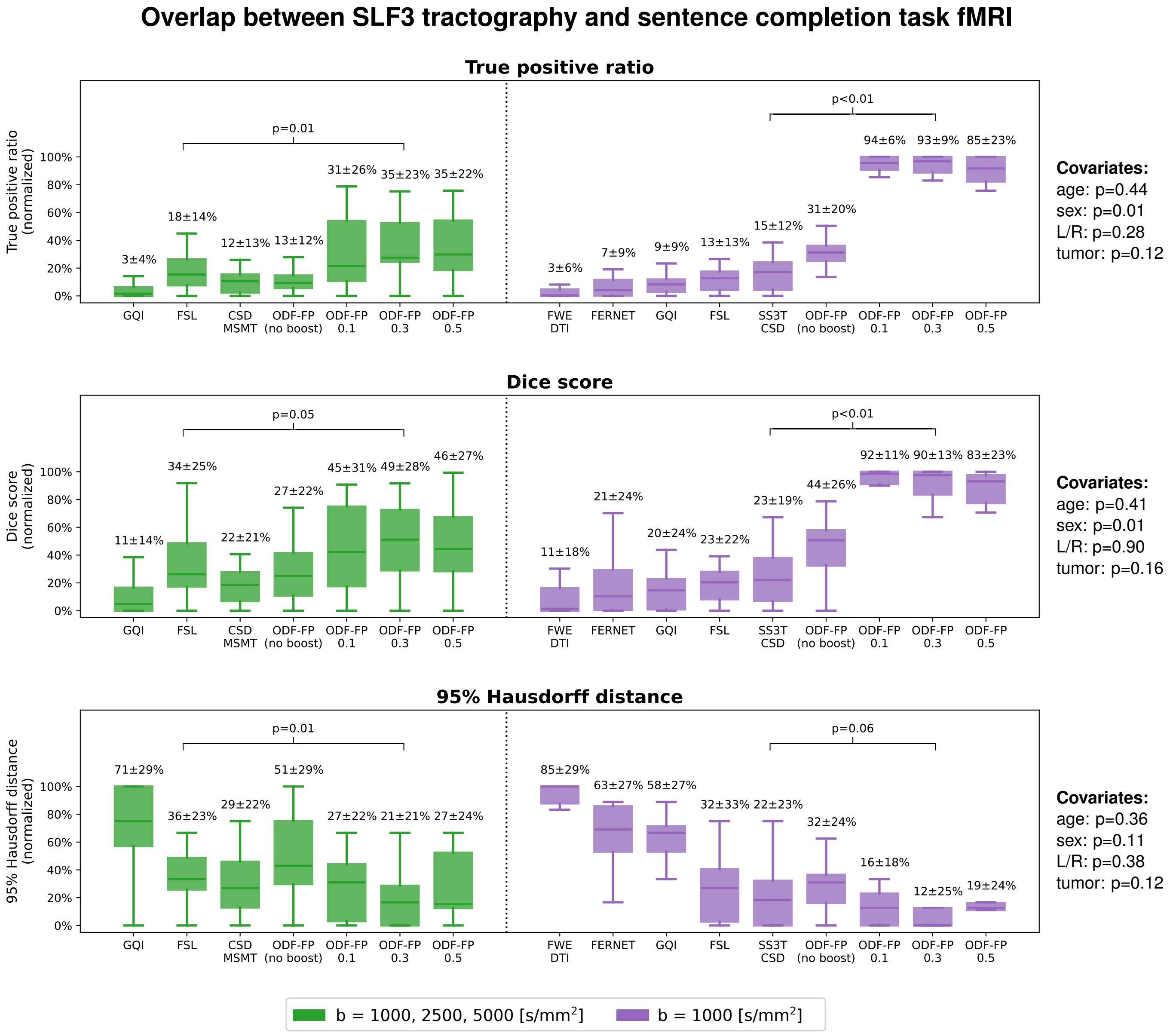

Supplementary Figure S8. Box plots of the relative overlap between Frontal Aslant Tract (FAT) and the cortical area activated during the sentence completion functional MRI (fMRI) task. Tractography was produced from the fully sampled (green) or the subsampled clinically-feasible diffusion MRI (purple). The mean and standard deviations of the normalized True positive rates, Dice scores, and 95% Hausdorff distances are given above the respective box whiskers. ODF-FP is compared with the best-performing reference method, i.e., FSL in the fully sampled and SS3T-CSD in the subsampled data set. The p-values of the covariates (patient's age and sex, tumor hemisphere and tumor type) are given on the right side.

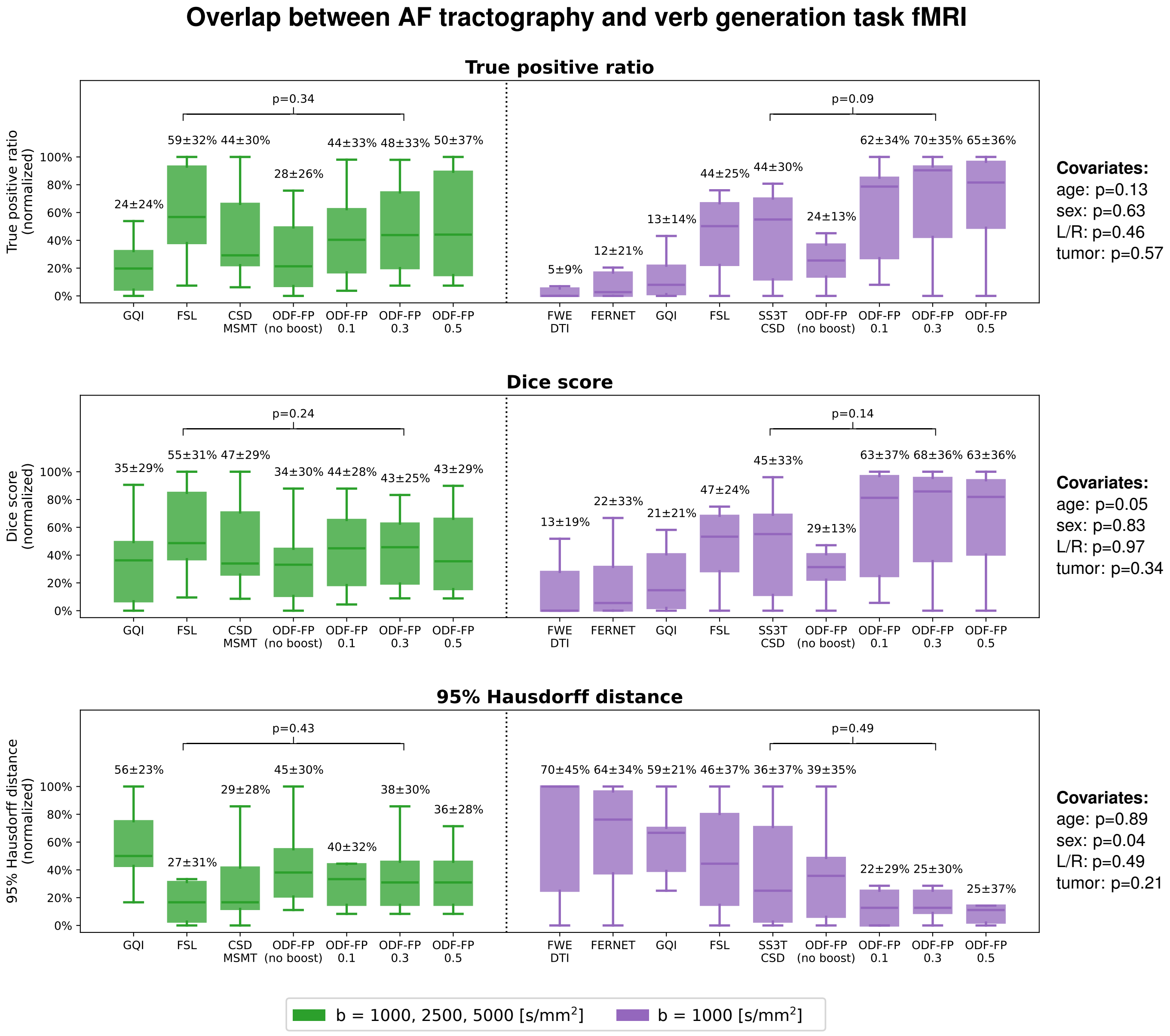

Supplementary Figure S9. Box plots of the relative overlap between Arcuate Fasciculus (AF) and the cortical area activated during the verb generation functional MRI (fMRI) task. Tractography was produced from the fully sampled (green) or the subsampled clinically-feasible diffusion MRI (purple). The mean and standard deviations of the normalized True positive rates, Dice scores, and 95% Hausdorff distances are given above the respective box whiskers. ODF-FP is compared with the best-performing reference method, i.e., FSL in the fully sampled and SS3T-CSD in the subsampled data set. The p-values of the covariates (patient's age and sex, tumor hemisphere and tumor type) are given on the right side.

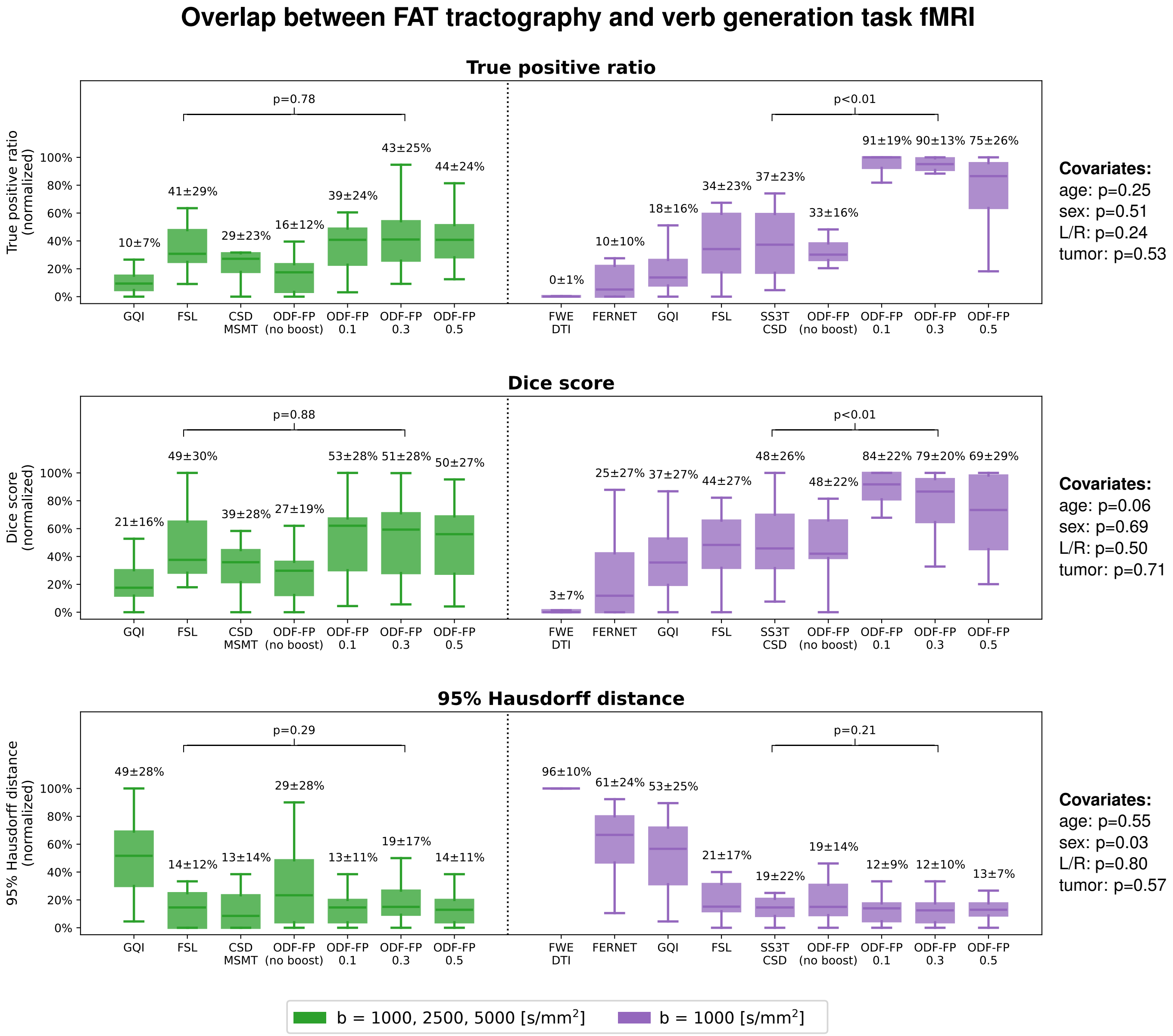

Supplementary Figure S10. Box plots of the relative overlap between Superior Longitudinal Fasciculus III (SLF3) and the cortical area activated during the verb generation functional MRI (fMRI) task. Tractography was produced from the fully sampled (green) or the subsampled clinically-feasible diffusion MRI (purple). The mean and standard deviations of the normalized True positive rates, Dice scores, and 95% Hausdorff distances are given above the respective box whiskers. ODF-FP is compared with the best-performing reference method, i.e., FSL in the fully sampled and SS3T-CSD in the subsampled data set. The p-values of the covariates (patient's age and sex, tumor hemisphere and tumor type) are given on the right side.

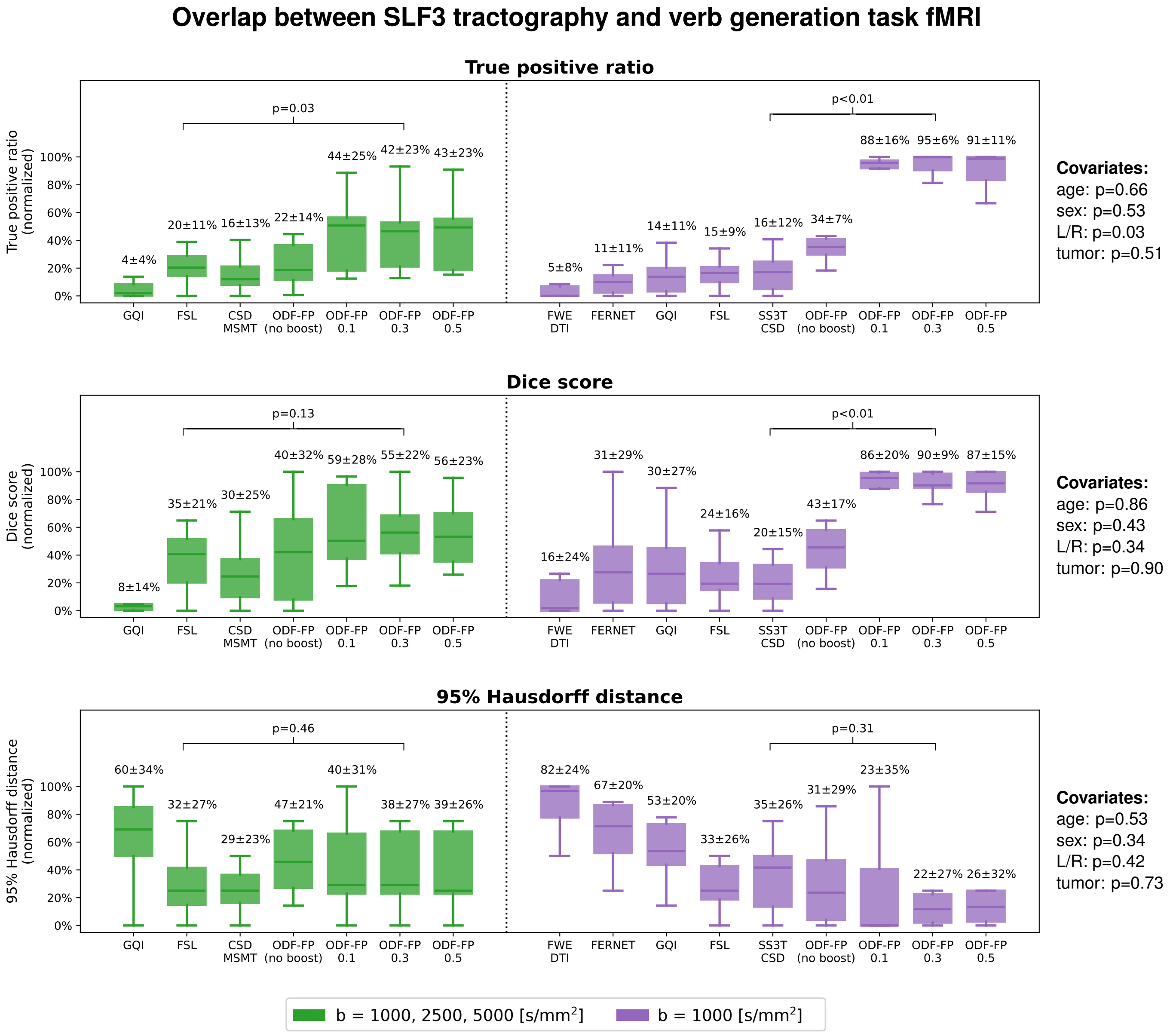

Supplementary Figure S11. Box plots of the relative overlap between Frontal Aslant Tract (FAT) and the cortical area activated during the verb generation functional MRI (fMRI) task. Tractography was produced from the fully sampled (green) or the subsampled clinically-feasible diffusion MRI (purple). The mean and standard deviations of the normalized True positive rates, Dice scores, and 95% Hausdorff distances are given above the respective box whiskers. ODF-FP is compared with the best-performing reference method, i.e., FSL in the fully sampled and SS3T-CSD in the subsampled data set. The p-values of the covariates (patient's age and sex, tumor hemisphere and tumor type) are given on the right side.

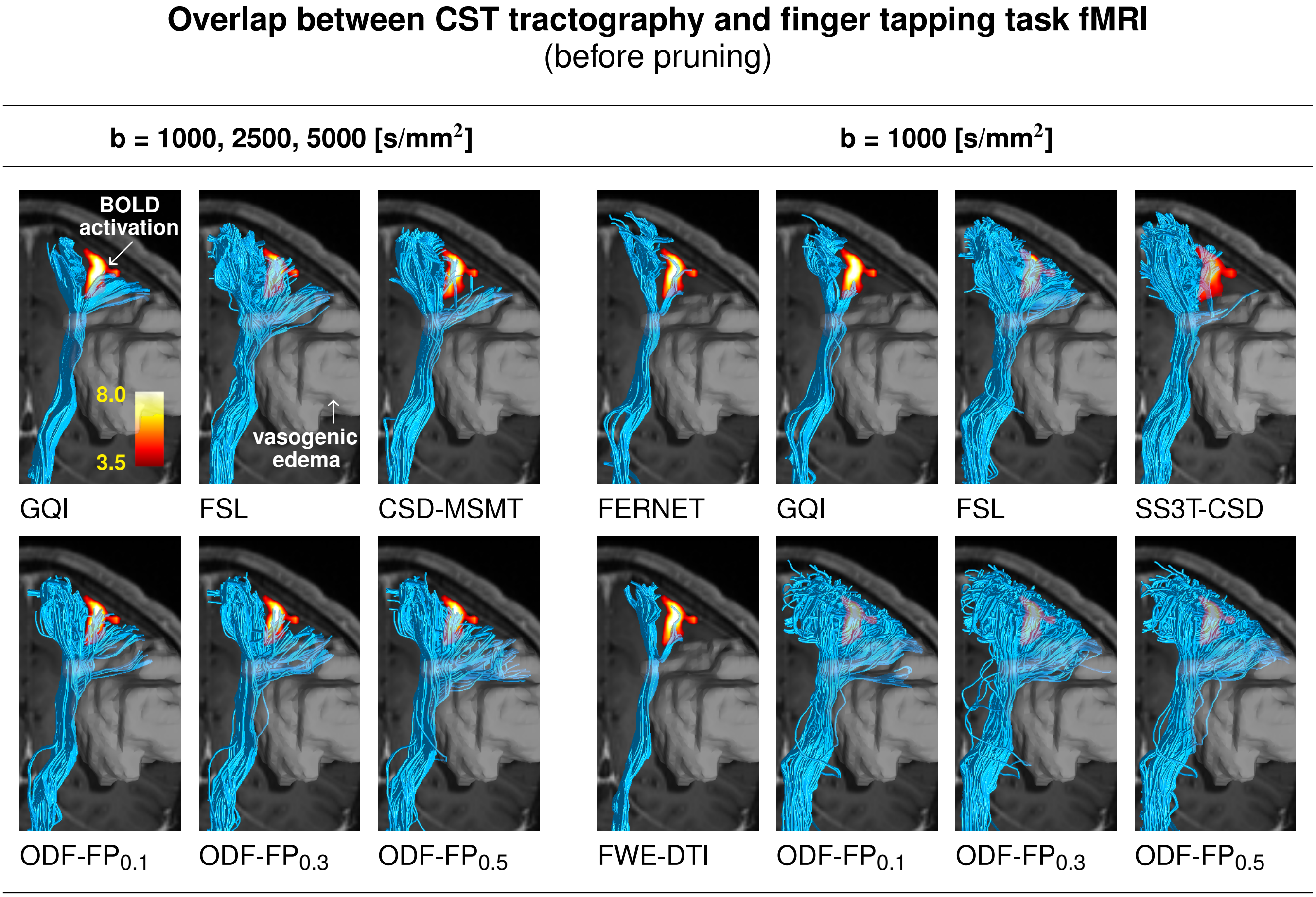

Supplementary Figure S12. Coronal view of Corticospinal Tract (CST) overlapping with the cortical region activated during the finger tapping functional MRI (fMRI) task. The tractography outcomes are presented as seen before manual pruning by a trained expert.

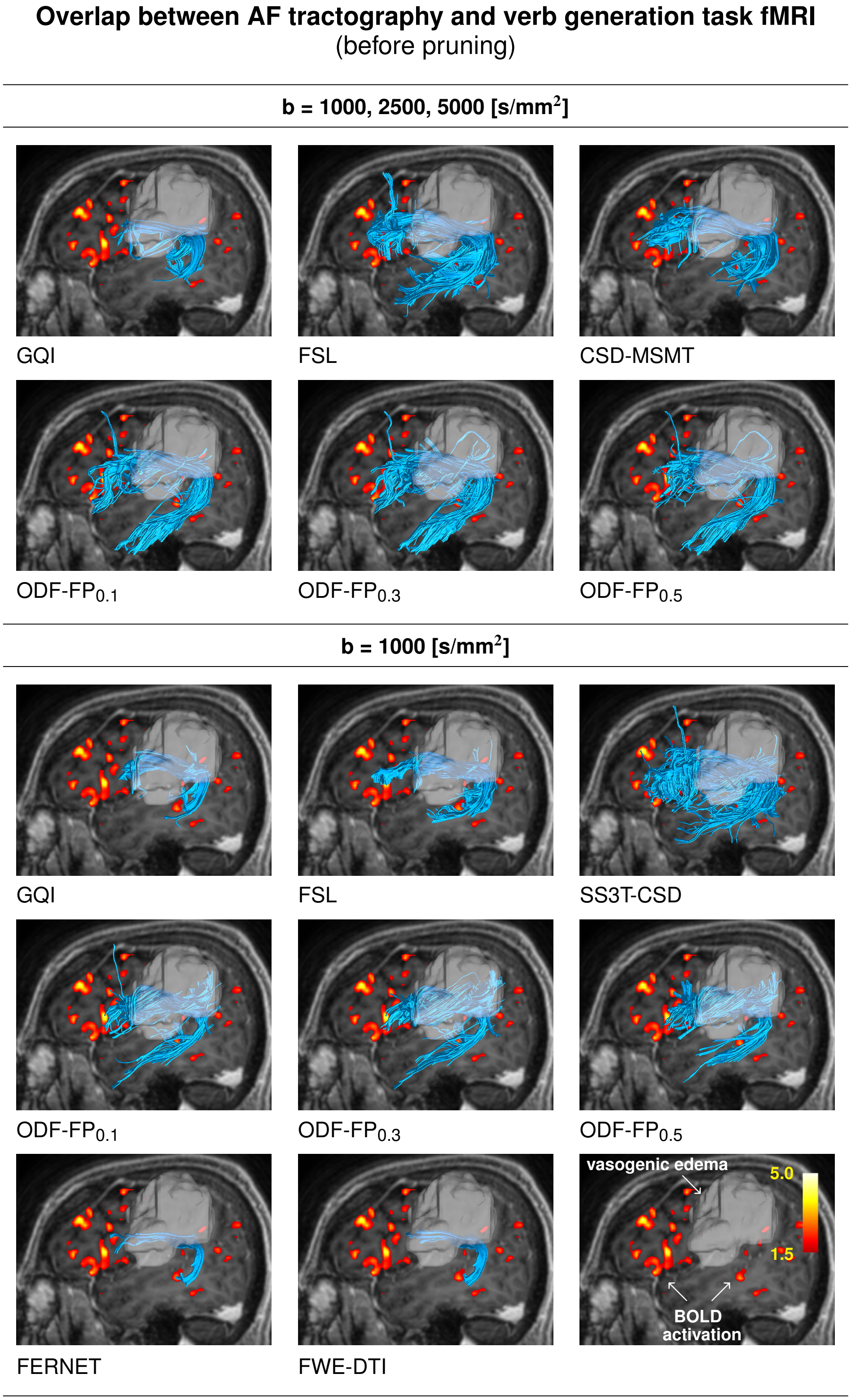

Supplementary Figure S13. Sagittal view of Arcuate Fasciculus (AF) overlapping with the cortical region (one representative slice overlaid as a heatmap) activated during the verb generation functional MRI task. The tractography outcomes are presented as seen before manual pruning by a trained expert.
